# Exploration of Circadian Rhythms in Patients with Bilateral Vestibular Loss

**DOI:** 10.1371/journal.pone.0155067

**Published:** 2016-06-24

**Authors:** Tristan Martin, Sébastien Moussay, Ingo Bulla, Jan Bulla, Michel Toupet, Olivier Etard, Pierre Denise, Damien Davenne, Antoine Coquerel, Gaëlle Quarck

**Affiliations:** 1 UNICAEN, COMETE, 14032 Caen, France; 2 INSERM, U1075, 14032 Caen, France; 3 Normandie Universite, Caen, France; 4 CHU de Caen, Service des Explorations Fonctionnelles, 14000 Caen, France; 5 Centre d’explorations fonctionnelles oto-neurologiques, 10 rue Falguière, 75 015 Paris, France; 6 CHU de Caen, Laboratoire de pharmacologie-toxicologie, 14000 Caen, France; 7 Theoretical Biology and Biophysics, Group T-6, Los Alamos National Laboratory, Los Alamos, New Mexico, United States of America; 8 Institut für Mathematik und Informatik, Universität Greifswald, Walther-Rathenau-Straße 47, 17487 Greifswald, Germany; 9 Department of Mathematics, University of Bergen, P.O. Box 7800, 5020 Bergen, Norway; CNRS, University of Strasbourg, FRANCE

## Abstract

**Background:**

New insights have expanded the influence of the vestibular system to the regulation of circadian rhythmicity. Indeed, hypergravity or bilateral vestibular loss (BVL) in rodents causes a disruption in their daily rhythmicity for several days. The vestibular system thus influences hypothalamic regulation of circadian rhythms on Earth, which raises the question of whether daily rhythms might be altered due to vestibular pathology in humans. The aim of this study was to evaluate human circadian rhythmicity in people presenting a total bilateral vestibular loss (BVL) in comparison with control participants.

**Methodology and Principal Findings:**

Nine patients presenting a total idiopathic BVL and 8 healthy participants were compared. Their rest-activity cycle was recorded by actigraphy at home over 2 weeks. The daily rhythm of temperature was continuously recorded using a telemetric device and salivary cortisol was recorded every 3 hours from 6:00AM to 9:00PM over 24 hours. BVL patients displayed a similar rest activity cycle during the day to control participants but had higher nocturnal actigraphy, mainly during weekdays. Sleep efficiency was reduced in patients compared to control participants. Patients had a marked temperature rhythm but with a significant phase advance (73 min) and a higher variability of the acrophase (from 2:24 PM to 9:25 PM) with no correlation to rest-activity cycle, contrary to healthy participants. Salivary cortisol levels were higher in patients compared to healthy people at any time of day.

**Conclusion:**

We observed a marked circadian rhythmicity of temperature in patients with BVL, probably due to the influence of the light dark cycle. However, the lack of synchronization between the temperature and rest-activity cycle supports the hypothesis that the vestibular inputs are salient input to the circadian clock that enhance the stabilization and precision of both external and internal entrainment.

## Introduction

Daily locomotion and postural regulation arise from a complex system where somatosensory, vestibular, and visual information is interrelated [1]. Disturbance or suppression of any of these processes leads to postural imbalance and impaired locomotion [2–4]. Semicircular canals and otoliths of the vestibular apparatus sense rotational and linear head movements respectively. Inputs are then projected to ocular and spinal motoneurons which generate compensatory eye (vestibulo-ocular reflex, VOR) and neck (vestibulo-colic reflex, VCR) movements in order to stabilize the visual field and posture [5,6].

In patients suffering from bilateral vestibular loss (BVL), clear disruptions of angular and linear VOR are present in low frequency movements [7–9], leading to oscillopsia and blurred vision. BVL patients often report gait ataxia, particularly at initiation [10,11], imbalance in dark or rough ground environments, unsteadiness, and abnormal head and body righting reactions [1,9,12,13]. Postural impairment is notably observed when other sensory inputs are manipulated [3,14].

New insights have expanded the influence of the vestibular system to other functions such as spatial orientation, since BVL in humans causes an alteration in internal spatial representation [15] and in animals results in poor performance in a spatial memory task [16,17]. Other results from BVL models in animals and humans have shown that the vestibular system influences autonomic functions such as cardiovascular [18,19] and bone regulation [20,21]. Recently, the involvement of the vestibular system in the regulation of circadian rhythmicity in animal models has emerged including a direct influence on the free-running circadian period [22]. Data have demonstrated some thermoregulation and circadian rhythm disruptions in wild type mice and rats during constant 2G gravity but not in mice (Ko *het* (-/-) mice) devoid of otoconia [23–25]. A total suppression of vestibular input by BVL in rats led to a sharp fall in core temperature and a switch to an ultradian rhythm in the days following the vestibular lesion [26]. Circadian rhythmicity is controlled by two fundamental components: (i) an endogenous biological clock located in the suprachiasmatic nucleus of the hypothalamus which generates circadian rhythms [27,28], and (ii) environmental factors such as day/night cycle [29,30] and non-photic time cues such as physical activity [31]. Together, these photic and non-photic time cues allow the biological clock to be synchronized with one's own environment. It is not yet known how physical activity influences the circadian system, but the vestibular system, which detects any type of movement, may be involved.

Functional links between the vestibular system and circadian rhythms have been described above, with neuro-anatomical pathways between the median vestibular nuclei and the suprachiasmatic nucleus [32]. These findings strongly suggest that the vestibular system constantly influences hypothalamic regulation of circadian rhythms on Earth in animals.

The aim of this study was to record data on human circadian rhythmicity in patients suffering from a bilateral vestibular loss, which has never before been considered. If the vestibular system influences regulation of circadian rhythms, we can hypothesize that BVL will lead to chronobiological disorders when compared control healthy participants.

## Materials and Methods

### Participants

The patient group was composed of 9 adults (5 males and 4 females, with a mean age of 53.6 ± 11.5 yrs) suffering from bilateral idiopathic loss of vestibular function (BVL) for 7 ± 4 years but not from hearing loss or associated neurological symptoms. None of the patients complained of persistent oscillopsia. The diagnosis was based on the absence of abnormal responses to a battery of neuro-otological tests performed in the “Centre d’Explorations Fonctionnelles Oto-neurologiques” (Paris, France) before inclusion in the study. Abnormal canal function was evaluated using a bithermal caloric irrigation for the right and the left ear at both 44°C and 30°C (caloric testing),and by measuring the vestibulo-ocular reflex VOR during a low frequency rotatory test (0.05 Hz, period 20 s). Absence of response or inferior to 5°/s for caloric testing and 12°/s was reported in BVL patients. The Video Head Impulse Test (VHIT, Otometrics, see Curthoys & Halmagyi, 1988) was performed with an automatic sensitive video camera system for each of the six canals. Moreover, the majority of these patients also had a deficit in high frequency tests with no compensatory eye movements and multiple catch up saccades. Otolithic (saccular) function was evaluated using cervical vestibular evoked myogenic potential (cVEMP) testing. Patients had no reponse to CVEMP or poor response with a weak amplitude (< 100 μV), characteristic of BVL [33]. The control group was composed of 9 healthy volunteers matched in age and gender, without any vestibular disease as confirmed by a caloric test, cervical VEMP, and subjective visual verticality [34].

All included participants came for a screening visit during which written informed consent was obtained after the study procedures were explained in detail. Self-evaluated chronotype was determined using the Horne and Östberg Morningness-Eveningness Questionnaire (MEQ). We excluded individuals with acute or unstable medical conditions, current or prior psychiatric diagnoses, current or prior shift work history neurodegenerative decease, alcohol or narcotic consumption and the use of any medications known to interfere with diurnal vigilance or sleep. This study received ethical approval by the ethics committee (CPP nord ouest III, ID: no. RCB: 2011-A001359-32), CHU Côte de Nacre, Caen, France. All participants provided written informed consent after the study procedures were explained in detail.

### General procedure

Four test sessions were organized, 2 for patients (*n* = 5 and 4) and 2 for healthy volunteers (*n* = 5 and 4). During each test session, participants first had a recording done of their habitual rest-activity rhythms at home for 15 days between May and July. None of the subjects were recorded during a holiday period or during work travel. None of the subjects declared crossing any time zone in the 6 months prior to the experiment. During the following week, they had a 24 h recording in our laboratory to record the circadian rhythmicity of core temperature, and salivary cortisol.

Rest-activity cycles: Participants were asked to wear an actigraph (Actiwatch^®^, Neurotechnology, Cambridge, UK) for the full 15 days except when showering or at the swimming pool. Using general recommendations of Ancoli-Israel [35], they were also asked to indicate their daily sleep schedule including bedtime and rising time, the estimated sleep onset and wake times, and their awakenings during the night.

Circadian rhythmicity: Participants stayed in our laboratory from 12:00 PM to 01:00 PM the following day during which they underwent test sessions set at 03:00 PM, 06:00 PM, 09:00 PM and 6:00 AM, 9:00 AM and 12:00 PM when saliva sampling was performed. Participant received a core temperature pill receiver and the pill was ingested in the presence of a physician after their arrival at the laboratory on the first day.

During the wake period, participants were exposed to a light intensity maintained between 150 and 200 lux at eye level (ambient light). Participants had at least 6 h of sleep and few ambulatory restrictions [36–38], but they were allowed only to take part in activities that did not involve any physical load or excitement which included reading, watching a movie (only during the day between noon and 5 P.M), or playing cards. Ergogenic and stimulant drinks (e.g. coffee and tea) were not allowed. After the 09:00 PM test session, each participant went to their sleeping room and was asked to go to bed at 11:00 PM, in complete darkness, and to get up at 05:00 AM for the 06:00 AM test session. They were allowed to drink only a glass of water [39,40]. Participants then ate a standardized meal at least 2 h before the 09:00 AM and 03:00 PM sessions [41].

### Data Collection

#### Rest-activity cycle

Actigraphy consisted of body movement recordings using an accelerometer (Actiwatch 7, Actiwatch^®^, Neurotechnology, Cambridge, UK). The actigraph was placed on the non-dominant wrist. Each movement was recorded and stored in memory within the actigraph device. The amount of time per 1-mintime period in which the level of the signal produced in response to movement was above a 0.01G threshold was recorded. Filters were set to a range of 3 to 11 Hz. At the end of the 15 days of recording, the data was recovered, transferred, and analyzed via sleep analysis 7^®^ software (Cambridge Neurotechnology, UK).

#### Saliva sampling

Saliva was collected at each test session (03:00 PM, 06:00 PM, 09:00 PM and 6:00 AM, 9:00 AM and 12:00 PM) using a universal sampling device, Salivette® (SARSTEDT AG & Co, Nümbrecht, Germany). Participants were not allowed to eat or drink (except water), smoke, or brush their teeth for 30 min prior to sample collection [37,42].

Participants remained seated 15 min before all session times. Saliva sampling was then conducted while participants were in a sitting position. For each sample, participants were asked to rinse their mouth with a sip of water, place the cotton inside of the cheek, and make a chewing motion to stimulate salivation. After 2 min, the roll-shaped saliva collector was placed in the appropriate tube. Each sample was then centrifuged until a minimum amount of saliva (1 mL) was collected, after which it was immediately frozen at -18°C.

Cortisol values were assessed by electrochemiluminescence “ECLIA” (COBAS®; Elecsys 2010 modular analytics, Cobas E411, Roche Diagnostics (Suisse) SA 6343 Rotkreuz; sensitivity 0.308 μg/dL, intra-assay coefficient of variation <7.1%, inter-assay coefficient of variation <6.1%). The assessing staff was blind with respect to the participants' identity and their diagnosis.

#### Temperature Data

A real-time telemetric data acquisition system using a disposable ingestible core pill (8.7 mm in diameter x 23 mm in length; VitalSense®, Mini Mitter Co. Inc., Bend, OR, USA) coupled with a small receiver (120 x 90 x 25 mm; 200 g) carried by the participant was used to monitor and collect temperature data. Temperature data were recorded every minute for a full 24 h [43].

### Data analysis and statistical methods

Due to a substantial deviation from the protocol (use of medication affecting vigilance), one participant in the control group was excluded from the data analysis. All statistical analyses were performed on the 8 remaining control participants.

#### Actigraphy analysis

Data from the Actiwatch were analyzed with Sleep Analysis^®^ software (V 7.0, Cambridge Neurotechnology Ltd., Cambridge, UK). The circadian rhythm of the rest-activity cycle was analyzed with the NPCRA (Non-Parametric Circadian Rhythm Analysis) function [44]. The first day of the recording period was not taken into account to avoid incomplete data. NPCRA allowed us to determine the estimated circadian rhythm parameters, acrophase (peak time of the rhythm), the peak-to-peak amplitude, and the MESOR (mean level of activity) of the 14 days of recording. The mean daily activity level (count/h) over 24 h, during the habitual wake period and the habitual sleep period, was calculated. The ratio between the sleep and wake period was then estimated. The mean activity index (% of activity per hour) over 24 h, during the wake and sleep period, was also calculated. These parameters were calculated for the whole week, and separately for the weekends and the weekdays.

Finally, we also considered two circadian sleep-wake rhythm parameters provided by the NPCRA analysis: interdaily stability (IS) and intradaily variability (IV) and L5 mid [45]. IS quantifies the strength of coupling of the sleep–wake cycle to the 24 h regularity in the environment. A low IS is indicative of a weak circadian rhythm. The IV quantifies the fragmentation of periods of rest and activity. A high IV is indicative of many transitions between periods of rest and activity [46]. The L5 mid corresponds to the central time of the least active 5h period (L5) in the average 24h pattern.

Sleep analysis software provided an actigraphic estimation of mean sleep latency (SL), sleep onset (SO), wake time, total sleep time (TST), wake time after sleep onset (WASO), and sleep efficiency (SE) for each participant. These parameters were calculated for the whole week period, the weekends, and the weekdays.

Sleep analysis was performed each night (except the first). A 12 h window was used to separate nocturnal sleep from wake periods. None of the participants stated that they had slept during the day. A 1 min epoch length and a medium setting of sensitivity was used, with an awake threshold of 40 counts per epoch, and SO was defined as the first period with a minimum of 10 min of consecutively recorded immobile data with no more than 1 epoch of movement within that time [47].

Sleep and actigraphy parameters were compared between the two groups using unpaired *t*-tests or a non-parametric Mann-Whitney test when data were unequally distributed (Sigma-Stat^®^). Weekday and weekend parameters were compared using paired *t*-tests. Potential heteroscedasticity was automatically taken into account by the testing procedure, which uses the Welch-test version of the t-test when indicated.

*p*-values of less than 0.05 were considered statistical significant.

#### Circadian rhythms analysis

For salivary cortisol, the preliminary analysis as well as visual inspection suggested the presence of a simple time trend, and potentially the presence of group effects. Therefore, we selected a linear mixed-effects model as our approach. In order to capture serial within-subject correlation and heteroscedasticity, we investigated different correlation and variance structures, respectively (see details in appendix). Following the identification of both a time trend and a group effect, we proceeded by testing the effect of time of day on salivary cortisol using a one-way repeated measures ANOVA, to identify daytimes for minimum and maximum values, with the factor “time” as the repeated factor [48–50]

A stepwise multiple comparisons Student-Newman-Keuls (SNK) procedure was selected as a post hoc analysis.

Visual inspection of the temperature data suggested that it followed the typical sinusoidal pattern, with potentially minor deviations from the common shape in the patient group. Therefore, we carried out a non-parametric analysis on all data recorded by telemetry to display the waveform of the temperature curve of both patient and control groups. The temperature curve was approximated by a smoothing-type approach, more precisely local polynomial regression (LOESS, see Appendix for details, in particular concerning the selection of the smoothing parameter). In addition, we estimated a classical cosinor type model via non-linear least squares regression analysis. This allowed us to determine the best fit of a combined 24 h period cosine function of the form:

$$COSINOR(t)=m+a∙cos\left(\frac{(t-p)∙\pi }{12}\right).$$

The parameters m, a, and p represent the circadian mesor, amplitude and acrophase respectively. Note that our setup implicitly assumes a fixed 24 h period of the rhythm. For both the estimated COSINOR functions as well as the alternative rhythm functions resulting from the LOESS approach, we determined confidence bands by a Monte-Carlo approach.

The estimated LOESS curves and the group-specific COSINOR functions suggested differences between controls and patients. To investigate the potential presence of group effects on circadian parameters of temperature, we proceeded by first fitting the COSINOR function without any effects via generalized non-linear least squares (gnls). We then included a group effect in the mesor, amplitude, and phase, for hypothesis testing (see the appendix for details).

Lastly, individual circadian rhythm acrophases were calculated using COSINOR on each participant curve and the interval between an individual's habitual SO and the temperature acrophase (phase angle) was calculated in this way.

The phase angle was compared between the two groups using unpaired *t*-tests or a non-parametric Mann-Whitney test when data were unequally distributed (Sigma-Stat^®^). F-tests were used to measure the variability of the acrophase for the participants of the two groups. Associations between variables were assessed using a Pearson moment product correlation (Sigma-Stat^®^). *p*-values of less than 0.05 were considered statistically significant.

## Results

### Rest-activity cycle

The relative amplitude of the circadian rhythm of the rest-activity cycle during the 15 days of actigraphy was damped in the patient group (0.86 ± 0.04 count/min) compared with the control group (0.93 ± 0.03 count/min; *p*<0.005). The circadian acrophase and mesor were similar in the two groups (Table 1). On average, patients were as active as the control group participants with a similar mean 24 h actigraphy (Patients 201 ± 44 count/min; Control 201 ± 49 count/min) and wake actigraphy (Patients 266 ± 66 count/min; Control 286 ± 61 count/min) during the full 15-day recording period. However, the patients showed more actigraphy during the sleep period (63 ± 23 count/min) compared to control participants (35 ± 23 count/min). The ratio between wake and sleep periods was thus significantly reduced in the patient group compared to the control group (*p*<0.05). Mean values are given in Table 2.

**Table 1:** Mean parameters of the circadian rhythms of the sleep wake cycle.

Sleep/Wake cycle(count/min)	Patients	Control	*p*
Acrophase (hh:mm)	3:05PM ± 1:03h	3:09PM ± 0:19h	0.84
Amplitude (count/min)	0.86 ± 0.04	0.93 ± 0.03	0.004*
Mesor (count/min)	193.11 ± 53.84	203.88 ± 45.6	0.68

Mean parameters of the circadian rhythms of the sleep/wake cycle calculated by the Non-Parametric Circadian Rhythm Analysis (NPCRA) method.

* indicates a significant difference with *p*<0.05.

**Table 2:** Mean actigraphy parameters (M±SD for the whole week, weekdays and weekends.

	Whole week			Weekdays			Weekends			Weekdays vs weekends	
	Patients	Control	*p*	Patients	Control	*p*	Patients	Control	*p*	Patients	Control
**Daily actigraphy (count/min)**	201±49	201 ± 44	0.98	189 ± 56	195 ± 40	0.84	202 ± 54	218 ± 76	0.65	0.03*	0.34
**Wake actigraphy (count/min)**	266 ± 67	286 ± 61	0.55	250 ±73	277 ± 60	0.43	276 ± 76	308 ± 98	0.50	0.04*	0.34
**sleep actigraphy (count/min)**	63 ± 23	35 ± 23	0.03*	63 ± 34	32 ± 17	0.04*	47 ± 20	41 ± 42	0.76	0.23	0.42
**wake/sleep actigraphy ratio**	5 ± 2	12 ±8	0.04*	5 ± 2	12 ± 9	0.06	7 ± 3	12 ± 9	0.15	0.09	0.99
**daily actigraphy index (%)**	66 ± 8	62 ± 4	0.17	67 ± 8	61 ± 4	0.07	63 ± 7	61 ± 6	0.57	0.001*	0.95
**wake actigraphy index (%)**	85 ± 10	82 ± 4	0.17	87 ± 10	82 ± 4	0.27	81 ± 9	78 ± 8	0.49	0.01*	0.32
**sleep actigraphy index (%)**	26 ± 8	21 ± 6	0.35	27 ± 9	21 ± 6	0.18	24 ± 8	20 ± 7	0.25	0.32	0.48

The mean daily activity level over 24 h, during the habitual wake period and the habitual sleep period, were calculated. The ratio between sleep and wake period was thus estimated. The mean activity index (% of activity per hour) over 24 h, during the wake and sleep period, were also calculated. These parameters were calculated for the whole week, and separately for the weekends and the weekdays.

* indicates a significant difference with p<0.05.

On average, patients and control participants were 86±10% and 85±4% active, respectively, during the wake period and 28±8% and 21±6% active, respectively, during the sleep period, without significant differences between the two groups.

A significant difference between weekdays and weekends could not be found for a mean 24 h wake and sleep actigraphy in control participants (see values in Table 2).

In contrast, the patient group exhibited a slight but significant (*p*<0.05) difference in mean 24 h (202 ± 54 count/min) and wake (276 ± 76 count/min) actigraphy during the weekend compared to the weekdays. Actigraphy during sleep was not significantly different between weekdays (64 ± 34 count/min) and the weekends (47 ± 20 count/min) in the patient group, but it was higher than in the control group (32 ± 16 count/min; *p*<0.05) during the weekdays. No difference between the two groups was reported during the weekends for actigraphy.

IV and IS parameters did not show significant differences between patients (IV = 0.85 ± 0.16; IS = 0.53 ± 0.08) and the control group (IV = 0.77 ± 0.16 *p* = 0.34; IS = 0.50 ± 0.11 *p* = 0.50). Values recorded for IV seemed to be higher in patients but did not reach statistical significance compared to the control group, possibly due to the low number of subjects. L5mid was also not significantly different between patients (3:412AM±1:06h) and the control group (3:30AM±0:45h; *p* = 0.69).

L5 (average activity) strongly tended to be higher in patients group (1368.57 ± 677.90) than in control group (772.38 ± 361.75; p = 0.054 Mann Whithney rank sum test).

### Nocturnal sleep

The 15-day actigraphy analysis did not show any significant difference between the 2 groups in TST, WASO SL, SO, or wake time values (Table 3). SE values tended only to be increased in the patient group (78.8% vs. 85.35% in control group, *p* = 0.06). WASO represents 15.12% of the patient’s TST vs. 10.14% in control group (*p* = 0.08). Interestingly, L5 negatively correlates with sleep efficiency values in patient group (r = -0.789; p = 0.035)

**Table 3:** Mean sleep parameters (M±SD) estimated by sleep analysis for the whole week, weekdays, and weekends.

Sleep Parameters (M±SD)	Whole week			Weekdays			Weekends		
	Patients	Controls	*p*	Patients	Controls	*p*	patients	Controls	*p*
Wake time (00:00)	07:16 ± 0:40h	07:47 ± 0:40h	0.18	06:56 ± 1:24h	07:44 ± 0:52h	0.19	07:10 ± 1:31h	07:53 ± 1:00h	0.28
SO (00:00)	0:00 ± 0:45h	23:55PM ± 0:53h	0.78	00:01PM ± 0:47h	23:55PM± 0:36h	0.75	23:58PM ± 0:53h	23:51PM ± 0:59h	0.82
TST (h)	06:16 ± 1:05	07:02 ± 1:11	0.22	06:14 ± 1:04	06:59 ± 1:08	0.22	06:22 ± 1:11	07:09 ± 1:23	0.27
WASO (h)	01:05 ± 0:27	00:50 ± 0:18	0.21	01:04 ± 0:26	00:49 ± 0:20	0.23	01:10 ± 0:32	00:51 ± 0:16	0.18
SL (h)	00:18 ± 0:13	00:12 ± 0:06	0.30	00:13 ± 0:10	00:13 ± 0:08	0.92	00:14 ± 0:07	00:09 ± 0:05	0.17
SE (%)	78.83 ± 6.86	85.35 ± 5.61	0.06	79.30 ± 6.53	86.05 ± 5.02	0.04*	77.66 ± 9.29	85.13 ± 7.69	0.11
bed time (00:00PM)	11:42 ± 0:52h	11:42 ± 0:39h	0.99	11:44 ± 0:54h	11:42 ± 0:33h	0.93	11:38 ± 0:56h	11:41 ± 0:59h	0.91
Rising up time (00:00AM)	07:34 ± 0:43h	07:59 ± 0:38h	0.24	07:31 ± 0:41h	07:52 ± 0:45h	0.35	07:44 ± 0:57h	08:14h ± 0:31	0.24

Sleep measurement for wake time, sleep onset (SO), total sleep time (TST), wake after sleep onset duration (WASO), sleep latency (SL), sleep efficiency (SE), bed time (00:00PM) and rising up time (00:00AM)

* indicates a significant difference with p<0.05.

Patients and control participants reported the same habitual bedtime (11:42 PM ± 0:57 h and 11:42 PM ± 0:39 h respectively); patients and control participants reported rising time at 7:34 AM ± 0:43 h and 7:59 AM ± 0:38 h, respectively. When the sleep parameters obtained during weekdays and weekends were compared, SE was significantly lower in the patient group (*p*<0.05). One may note that SE during the weekdays was similar to that observed during the whole week in patients, but this result could confirm the tendency observed between groups for SE during the whole week. No differences were observed between the groups for bedtimes, rising times, SO, wake time, TST, and WASO values, during weekends or weekdays (see Table 3).

### Salivary cortisol

**Fig 1:**
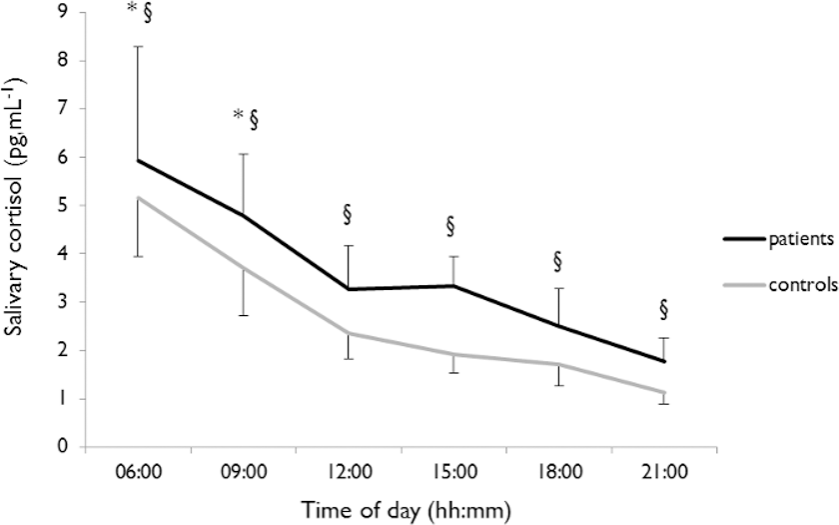
*Salivary cortisol levels*. Salivary cortisol profiles are shown for Patients (black) and Controls (grey). * indicates a significant effect of time of day. § indicates a significant difference between groups.

On average, both groups displayed similar profiles of salivary cortisol, but the mean cortisol level was significantly higher in the patient group (3.49 ± 1.85 pg/mL compared to 2.68 ± 1.55 pg/mL^;^ *p* = 0.02) and the patient group presented a higher cortisol value than the control group at all times of day (*p* = 0.001; Table 4). Higher values were recorded during the 6:00 AM session test (5.95 ± 2.17 pg/mL for patients; 5.17± 1.22 pg/mL for controls; *p*<0.001) compared to other session tests, and lower values were reported during the 12:00, 3:00, 6:00 and 9:00 PM session tests (Fig 1).

**Table 4:** Estimated parameters (intercept and slope) of salivary cortisol for control and patients.

Type of model and parameters subject to a session effect	df	AIC	BIC	loglik	Test	LRT	*p*
1. Null model (m_o_) Group effect: none	6	243.21	258.47	-115.61	1 vs. 2	88.06	
2. Selected model (m_s_) Group effect: phase	7	233.60	251.40	-109.80	2 vs. 3	11.62	**<0.01**
		Value	SD	t-value	*p*-value		
Intercept	Control	4.90	0.29	17.03	< 0.001		
	Δ patients	0.74	0.18	4.13	0.001		
Slope	Control& patients	-0.18	0.015	-12.49	<0.001		

The columns show (from left to right) the estimated parameter value, the standard deviation of the estimate (SD), the t-statistic, the t-value and the resulting p-value.

In order to take varying variability into account, based on AIC and BIC we selected a power variance function with different coefficients for the treatment and control group. The presence of residual within-subject autocorrelation was not supported.

In order to determine the best model fitting the data, we first built a model with constant intercept as basic model (m_0_). Then, we added step-wise a simple time trend, a group effect on the intercept, a group effect on the slope, and a group effect on both. The finally selected model (m_s_) has common slope for both treatment and control group, but varying intercept. Selection was supported by AIC, BIC, and LRT, which all showed clear preference compared to a simple linear regression (m_0_). The slope of 0.18 means that slivary cortisol was lowering by 0.18 pg/mL per hour

The salivary cortisol diurnal rhythm of the patient group tended (*p* = 0.07) to be delayed, with a mean acrophase of 7:14 AM ± 1:54 h, relative to that of the control group estimated at 5:46 ± 1:03 h (Table 5). One patient did not display circadian rhythms of cortisol. The estimated amplitude was similar in both the patient (3.76 ± 1.43 pg/mL) and the control group (3.63 ± 0.73 pg/mL). The MESOR tended (*p* = 0.053) to be higher in the patient group (3.64 ± 0.93 pg/mL) than in the control group (2.89 ± 0.38 pg/mL).

**Table 5:** Mean parameters of the circadian rhythms of the salivary cortisol.

Cortisol (pg/mL)	Patients	Control	*p*
Acrophase (hh:mm)	7:14AM ± 1:54h	5:46AM ± 1:03h	0.07
Amplitude (pg/mL)	3.76 ± 1.43	3.63 ± 0.77	0.82
Mesor (pg/mL)	3.64 ± 0.93	2.89 ± 0.38	0.053

Mean parameters of the circadian rhythms of salivary cortisol calculated by the COSINOR method.

* indicates a significant difference with p<0.05.

### Temperature

**Fig 2:**
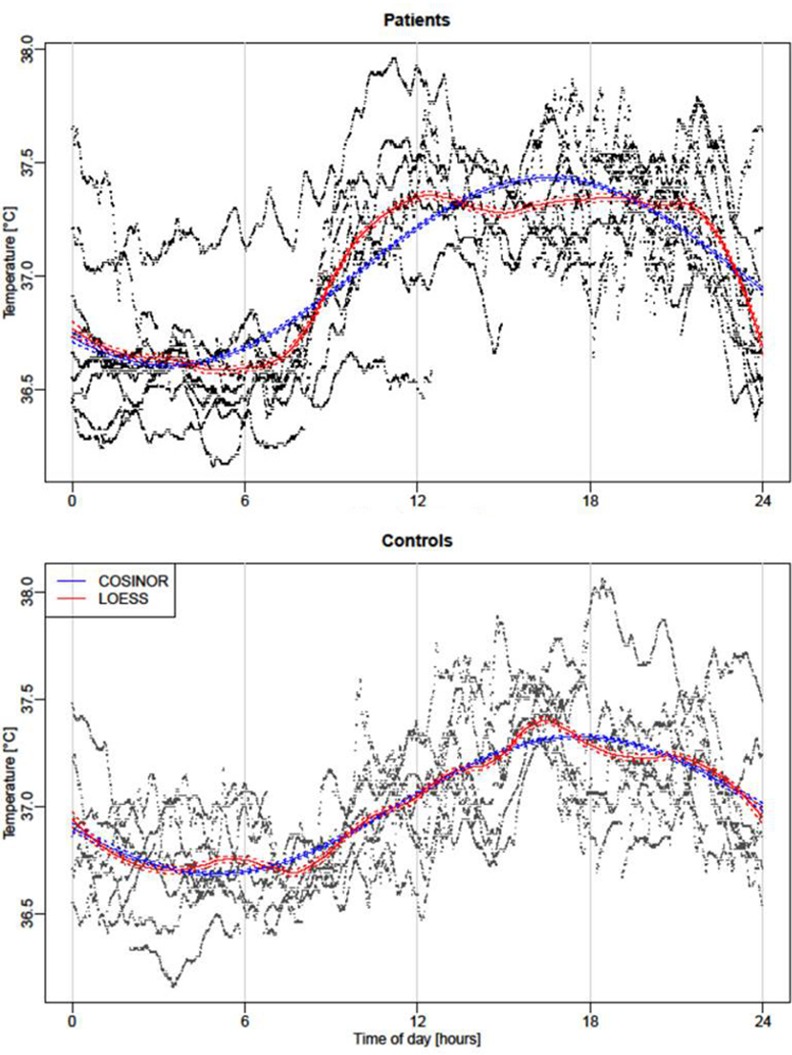
*Body Temperature curves*. *Temperature COSINOR curves (blue) and smoothed curves by local polynomial regression (function “LOESS” in red) for Patients (upper pattern) and Control group (lower pattern). The solid blue lines corresponds to results from the estimated cosinor function The dashed lines around the solid line correspond to a confidence band of level* $100\times \sqrt{0.95}\%$ *for the estimated cosinor curve. Solid red lines result from local polynomial regression (with span parameter α* = 0.25). *As before, dashed red lines correspond to the borders of the confidence band of level* $100\times \sqrt{0.95}\%$. *Thus, areas in which the two confidence bands do not overlap indicate a difference between the two functions with confidence level 95%. The highest and lowest values estimated by the LOESS method are respectively observed at 4:29 PM and 7:40 AM (although a nearly equally low minimum is already attained much earlier in the morning). The deviations were marginal since the local smoother mostly overlaps the COSINOR modeling only around 5:00 AM and 8:00 AM and around 4:00 PM and 7:00 PM. More precisely, the local smoother indicates that the temperature remains at a lower level than the COSINOR in the morning, with the largest difference attained at about 6:30 AM. In the following hours, the temperature rises faster than the COSINOR captures, and attains its steady state about 12:40 PM. In the following hours, the temperature remains relatively stable at a high level, roughly between 37.25 and 37.35°C. Then, in the evening at about 9:10 PM, the temperature drops, and the decline is stronger than the COSINOR is able to capture.*

The LOESS polynomial analysis first allowed us to investigate the characteristic of the overall waveform of the temperature curves of patients and controls. For the controls, the LOESS polynomial analysis showed that the overall temperature rhythm had a classic sinusoidal shape with a progressive rise in the morning and afternoon, and a fall in temperature in the early and late evening. Thus, the classic COSINOR mathematical method seems to provide an overall satisfactory fit of temperature data (Fig 2). The patients presented a different waveform than the control group, characterized by 2 large plateaus during the day and night separated by an early quick rise in temperature in the morning and a late quick fall in the evening.

**Fig 3:**
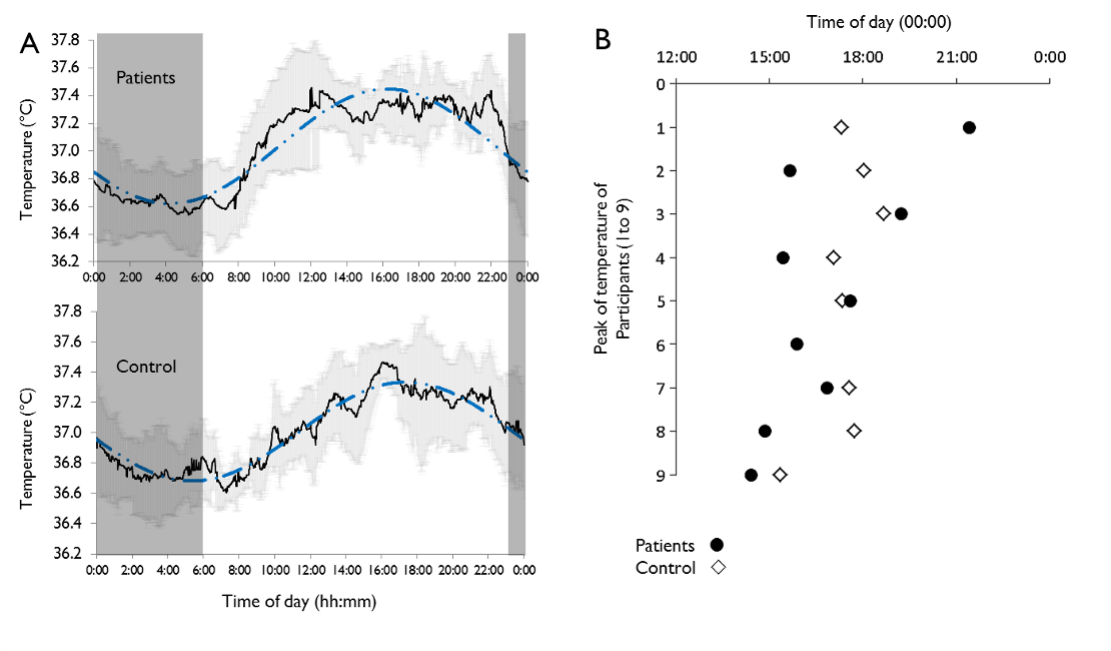
Body temperature rhythms and phase. *(A) Circadian rhythm of gastrointestinal temperature: Mean values (-) and SD (grey line) recorded every 60 s is shown for Patients (upper panel) and Controls (lower panel). The dashed blue lines represent modeling using the COSINOR method. Dark frame represents the sleeping period in the laboratory. (B) Individual acrophases of temperature in Controls (◇) and Patients (●). The 6^th^ control participant was removed from the analyses. The F-test revealed a larger variability (p<0.05) in the time of acrophase in Patients compared to Controls*.

Thus both patients and control participants displayed thus a diurnal temperature rhythm in accordance with the literature, with higher values in the afternoon. When COSINOR analysis was applied to all temperature data, the estimates confirmed the visual impression (see Fig 2): both curves did not differ strongly, but the acrophase was subject to a group effect (see Table 6). The estimated acrophase of the control group had a value of 17.49 (i.e., 5:29PM), whereas the acrophase of the patient group was significantly advanced by 1.22 (i.e., 1 h 13 min), in patients (4:16 PM). The comparison of individual circadian parameters revealed a larger variability of observed acrophase (Fig 3) in the patient group compared to the control group, with individual acrophases ranging from 2:24 PM to 9:25 PM (F test; *p*<0.05).

**Table 6:** COSINOR analysis performed on all data for each group.

Type of model and parameters subject to a session effect	df	AIC	BIC	loglik	Test	LRT	*p*
1. Null model (m_o_): COSINOR Group effect: none	5	-106533.0	-106493.4	53271.49			
2. selected model (m_s_): Extended COSINOR Group effect: phase	6	-106536.6	-106489.1	53274.32	1 vs 2	5.661363	**0.017**
**Parameters of the best model for temperature rise**				**Value**	**SD**	**t-value**	***p***
Mesor				36.98	0.02	1830.09	<0.001
Amplitude				0.68	0.024	14.57	<0.001
Acrophase				17.49	0.37	47.53	<0.001
Δ Acophase patient vs. control				-1.22	0.51	-2.40	0.016

The table displays data from the controls and patients. In order to investigate the potential presence of group effects, we fitted a cosinor function without group effect (i.e., the entire data). Inclusion of a group effect in the mesor, amplitude, and phase showed that a group effect in the phase is supported by a LRT (and AIC; but not BIC). Model m_0_ corresponds to the simple cosinor, m_s_ cooresponds to a cosinor with group effect in the phase:

The parameters m, a, and p correspond to mesor, amplitude, and acrophase respectively. Δ Acrophase re present the shift of the acrophase of the patient group, The estimated acrophase of the control group takes value 17.489 (i.e., 17h29), whereas the acrophase of the patients is significantly advanced by -1.22 (i.e., 1h13).

### Timing between circadian rhythms and sleep

Within the patient group, we noted 3 neutral, 4 moderate morning, 1 moderate evening and 1 extreme morning self-evaluated chronotypes. Control subjects rated themselves as neutral chronotypes. The Horne-Östberg MEQ is a reliable tool to describe the diurnal preference and the obtained score has been shown to be highly correlated with more objective circadian phase parameters, such as temperature [51,52]] DLMO [53].

We noted a discrepancy in patients between the estimated acrophase of temperature rhythm and the hypothetical time window in which it should appear according to the self-evaluated chronotype. This was confirmed by the absence of a correlation between the phase of temperature and SO (r = 0.04; *p* = 0.9). Similar to temperature, salivary cortisol presented a high inter-individual variability in the circadian profile for patients.

**Fig 4:**
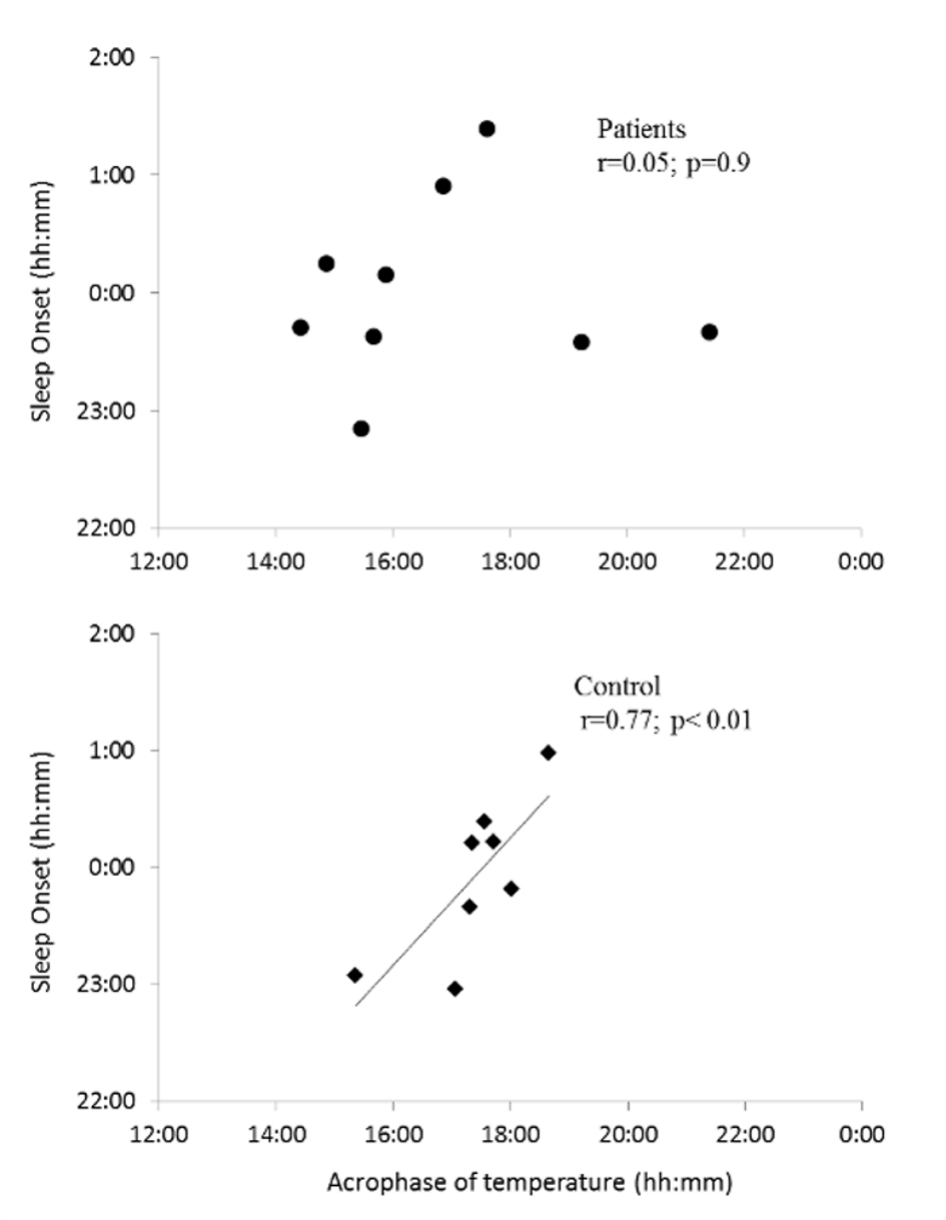
Timing between circadian rhythms and sleep. *Association between the timing of acrophase of temperature (h) and sleep onset (h) recorded during the 15-day actigraphy in Patients (upper panel) and in Controls (lower panel) groups*.

In contrast, in control subjects, the core temperature acrophase occurred during the expected hypothetical time window and the phase of the temperature circadian rhythms was significantly and positively correlated with SO (r = 0.77; *p*<0.05) (Fig 4).

Interestingly, the phase angle between temperature and habitual SO tended to be larger in patients (7.19 ± 2.36 h) than in the control group (6.55 ± 0.61 h), but this did not reach statistical significance (*p* = 0.07).

In the control group, the the cortisol peak occurred 2.03 ± 1.50 h after wake time (estimated by actigraphy) and was significantly (*p*< 0.01) greater than in the patient group, where wake time occurred by 0.04 ± 1.98 h (*p* = 0.95) after the cortisol peak time in patients, with 4 patients displaying their wake time before their cortisol peak.

## Discussion

The aim of this study was to provide a chronobiological profile of patients suffering from long-term bilateral vestibular loss. To date, the influence of the vestibular system on circadian rhythms has only been highlighted in rodents, and mainly used 2G hypergravity which caused disruption of circadian rhythms of temperature and locomotor activity [23–25]. Recent data has shown a similar disruption of circadian rhythms in rats, through chemical induction of a bilateral vestibular lesion [26]. Based on these findings, our hypothesis was that chronobiological disorders could be expected in patients suffering from bilateral vestibular loss. First, we decided to characterize the rest-activity cycle in this specific population in free living conditions using an actigraph in the patient’s home. Actigraphy has become a very reliable tool for exploring rest-activity cycles in field conditions [35,54]. Wrist actigraphy has already been used in specific populations such as elderly persons [55,56], hospitalized patients after surgery [57–59], or in clinical research studies [60,61], although the results must be carefully considered [62]. Differences in sleep habits between weekdays and weekends may lead to an irregular sleep/wake timing associated with sleep debt during the week as has previously been observed in healthy volunteers [63–65] or in shiftworkers [66]. However, in this study patients kept regular sleep/wake habits during weekdays and weekends. IS and IV values recorded for the patient group were similar to those reported in a previous study for insomniac people [46]. This study did not find differences between insomniac people and healthy subjects, meaning that altered circadian rhythms could not be a systematic feature in patients presenting sleep trouble. Fragmentation of the rest activity cycle (IV) seems to be higher in Patients but did not reach statistical significance compared to the control group, perhaps due to the low number of subjects.

Patients exhibited a “normal” amount of wake-time activity, reflecting their ability to complete their daily routine (e.g. office work, business travel, domestic activities, physical activities) despite their pathology. Symptoms such as gait ataxia and postural balance are commonly reported in patients [12] and may lead to anxiety-producing situations, decreased daily activity, and higher fatigability. In this study, patients had been diagnosed several years before, meaning that some dynamic compensatory processes had long been completed [14,67], allowing for normal postural adjustment in common situations. This could explain why patients in our study maintained an identical daily routine as the control group during the 15 day home actigraphy.

Interestingly, patients showed a higher amount of sleep time activity during the full week and during weekdays compared to healthy volunteers, which led to the observed damped amplitude of the rest-activity cycle and a strong tendency to higher movement during the L5 period, commonly associated to the trough of the sleep wake cycle. A damped amplitude of the rest-activity cycle constitutes a marker of disrupted sleep in a population, as has been noted in a population commonly suffering from sleep disorders [35]. It is also possible that maintaining daily activity to ensure effective working activity would be more difficult for these patients with bilateral vestibular loss. This pathology could disrupt their rest-activity cycle, explaining the higher nocturnal actigraphy recorded during weekdays but not during the weekend. Anxiety-like behavior is often described in this type of population [68], notably in multiple task situations wherein body movements or orientations are required. Moreover, bilateral vestibular loss seems to cause greater muscular fatigue, notably in cervical muscles, since Vestibulo colic reflexes do not work properly, leading to a noxious muscular compensation. The correlation between SE and L5 value supports the hypothesis that the relatively low SE can be related to the higher nocturnal sleep activity, during weekdays and the whole week, recorded in this group. SE recorded in patients was similar to that observed in specific populations presenting sleep disorders, such as elderly persons [69] or patients with insomnia [46,70]. Polysomnography would be necessary to clarify sleep parameters, as we know that actigraphy tends to overestimate TST and SE [35,61].

Total sleep time was not found to be significantly diminished in patients compared to healthy participants, although it appeared to be short (~6h) when we refer to the National Institute of Sleep and Vigilance. One hypothesis for this diminished sleep could be related to the postural perception of the body, since the supine position is commonly associated with sleep-promoting factors [71], e.g. decreased core temperature, and the effect of melatonin on increased heat loss [72,73]. The vestibular system has been shown to be actively involved in blood flow [74] and changes in cardiovascular function regulation [75], which are crucial for increased dissipation of heat. Although deficits in correcting blood pressure following vestibular lesions diminish over time in animals [75], we could hypothesize that the abnormal perception of gravity and posture caused by BVL might thus lead to some discomfort and cause diminished efficiency of sleep-promoting mechanisms. The cause of this chronodisruption could just be a masking effect on the sleep-wake cycle due to that discomfort. More research is needed to complete this study using a constant routine procedure with precise melatonin measurements to fully explore the effect of vestibular system pathology on the SCN. In the second part of the present protocol, participants were awake during the day, exposed to constant room light, and were allowed to sleep at night. The variation in the core temperature of the patient group revealed that long-term vestibular deficiency does not impair circadian regulation of temperature levels. This observation is not unexpected, since all patients have been living according to a regular sleep/wake cycle since the beginning of their pathology (several years). The visual system helped these patients substantially during their daily postural regulation and locomotion, the latter being the main transmitter of information related to the light/dark cycle directly to the SCN [76,77]. Moreover, previous studies showed that circadian waveform was restored in rats one week after induction of a vestibular lesion and continued one month after vestibular loss [26] and chronic 2G centrifugation [23]. Previous data in microgravity highlighted a damped temperature amplitude in astronauts during space flight, perhaps due to the 0G environment or the absence of the geophysical LD cycle in space [78]. The marked amplitude observed in our study could support this hypothesis where compensatory mechanisms [67] and long-term exposure to a regular light/dark cycle could have restored circadian rhythmicity in patients several years after their vestibular loss.

We noticed a particular shape of the temperature curve in the patient group, and the LOESS method confirmed that the patients presented relatively strong deviations from the classic sinusoidal curve of temperature, with an earlier and quicker rise in temperature soon after wake time and with a later, more abrupt decrease close to bedtime (see Figs 2 and 3). Thus, the temperature diurnal rhythm would be more likely influenced by the effects of the sleep-wake cycle upon the circadian rhythm in patients. Altered motion perception during the wake period in these people could disturb temperature regulation by the body clock; although we do not know the long-term effects of vestibular lesions on existing polysynaptic vestibulo-hypothalamic connections [32].

The temperature acrophase occurred in the expected time window according to the self-evaluated chronotype of the control group [51,52,79]. The fact that we only selected control participants presenting a neutral chronotype avoided any possibility of correlating core temperature and chronotype. However, an interesting finding in our study was that the large discrepancy in individual temperature acrophases observed in the patient group did not correlate with sleep habits, unlike that of healthy subjects. Such intra-group variability of circadian rhythms has already been observed in a specific population presenting sleep disorders [80]. Patients in our study presented a misalignment between phases of temperature rhythm and habitual sleep, as observed through actigraphy. This misalignment means that sleep onset cannot always be associated with an evoked temperature-lowering effect in patients, thus affecting sleep quality. The significant phase advance of temperature in the patient group, without phase advance of the sleep-wake cycle, confirmed this misalignment. Thus, in most patients studied in our laboratory, the fall in temperature occurred too soon in the afternoon, explaining why most patients complained about fatigue and sleepiness in the afternoon, with difficulty focusing on tasks at the end of their workday. In contrast, body temperature rose too early in the night, affecting the ability to sustain sleep effectively, possibly leading to sleepiness during the day and higher fatigue upon completion of daily tasks at work.

The lack of timing and correlation between self-rated chronotypes and peak temperature in patients leads to 3 different assumptions. First, the vestibular pathology itself and the lack of information related to movement could disrupt the stability of the circadian rhythm timing regulation, since vestibular information is currently sent to the central clock through the IGL [32]. Secondly, the daily discomfort encountered in patients due to possible poor balance or oscillopsia forces them to pay constant attention to their movements during their daily activities, which can cause general fatigue during the day and thus biased their self-evaluated morningness-eveningness preferences. Finally, one possible explanation comes from the reliability of the core temperature as a precise marker in this specific type of population. As indicated above, further research using a constant routine procedure (avoiding possible masking effects on temperature waveform) with precise melatonin and temperature measurements [81] are needed to confirm our hypothesis of an effect of the vestibular system on the circadian timing system.

The cortisol acrophase usually occurred within 2 hours before wake time [82], which was consistent with the results observed in the control group but not in the patient group. Half of the patients tested for cortisol displayed an internal desynchrony with a later peak of cortisol than that of the wake time. Patients and the control group displayed a classic pattern of salivary cortisol throughout the day. Higher cortisol levels observed in patients, particularly in the evening (9:00 PM), could lead us to suppose that salivary cortisol secretion could be delayed, confirming the tendency for the delayed acrophase of salivary cortisol observed. This observation could be linked to disrupted sleep, which can lead to higher levels of stress.

Due to the rarity of the bilateral vestibular decease (120/100,000 individuals;[83]), we cannot exclude that our study suffers from statistical limitation. Although we found strong tendencies for cortisol MESOR and acrophase, or for sleep parameters for example, we cannot exclude that the poor small number of subjects did not allow us to observe other significant results in this study. However, this number was sufficient to observe a significant result on actigraphy and circadian rhythms of temperature.

To conclude internal desynchronization in patients affecting circadian rhythms, sleep timing, grip strength, and hormonal secretion has been observed in other situations, wherein conflicting time cues such as bright light exposure during the night are given [84]. In this study, patients have suffered from idiopathic vestibular loss for several years and have developed some compensation mechanisms allowing them to perform their daily routine under the influence of the LD cycle. This explains why we observed a marked circadian rhythmicity in these patients. However, the relative desynchronization observed could be related to the lack of perception of acceleration of the head during the daily active phase. Although this study does not demonstrate a direct influence of the vestibular system on the clock, the loss of vestibular senses could thus disrupt the stabilization and the precision of the entrainment of circadian rhythms to the LD cycle and create some discomfort during the night. Our hypothesis is that the vestibular system could normally act as an actimeter, providing information of motion during the wake period. Our findings are consistent with, but also expand upon, previous work in vestibular-deficient mice, and continue to support the hypothesis [22] that vestibular inputs to the circadian clock may represent key sensory inflow for animal movement. Hence, vestibular inputs may represent salient input to the circadian clock that enhances the stabilization and precision of both external and internal entrainment.

Further studies including constant routine are necessary to properly highlight links between vestibular physiology and biological rhythms, but can be biased in these patients because of intrinsic masking factor, which is anxiety and stress. For this reason, we plan to explore circadian rhythms in constant routine condition in healthy subjects and to test the effect of vestibular stimulation on circadian rhythms amplitude and phase. This project will allow us to more deeply explore the mechanism linking vestibular physiology and biological rhythms.

## Appendix

In the following, we provide details on the statistical methods applied for cortisol and temperature data. All analyses were carried out using the software R 3.1.2 (www.r-project.org).

Cortisol analysis: Cortisol data were analyzed by linear mixed effects models as a modeling approach. In order to capture serial within-subject correlation and heteroscedasticity, we investigated different correlation and variance structures, respectively. No significant residual correlation was found, but a group and time-varying variance function was strongly supported. To evaluate whether a variable was significant, we followed the common approach of Pinheiro and Bates ([85]], p. 19 ff. and 83 ff.). That is, we compared models with and without the respective variable or effect, and compared them by means of a likelihood ratio test (LRT). Additionally, the model selection criteria AIC (Akaike information criterion) and BIC (Bayesian information criterion) were taken into consideration. When the LRT indicated a significant effect of a variable, the coefficients of the model were further examined and represented as the *t* and *p* values associated with each variable tested.

Temperature analysis: Smoothed curves were estimated via the LOESS approach (function “loess” in R), which stands for “LOcally wEighted Scatterplot Smoothing”. The principle idea of this method is the calculation of a smoothed value at any given point x by fitting a quadratic least squares regression to the data points in the neighborhood of x. It is noteworthy that the LOESS method requires the selection of the smoothing parameter (span) with a default value of 0.75. We selected this parameter by comparing the LOESS curve with the cosinor curve for the control group; fixing it at 0.25 resulted in both curves coinciding over almost the entire 24 h period. Then, the same span value served for analyzing the patient data, which revealed significant differences between the curve in terms of non-overlapping confidence bands for the estimated LOESS and cosinor curve.

For hypothesis testing, cosinor curves were estimated by generalized non-linear least squares (gnls). This approach allows specifying different residual correlation structures, which is important for inferential procedures. Using AIC and BIC as selection criteria, we chose the time series typical AR(1) form. In order to account for missing values, we estimated the AR(1) coefficient on all complete subsequences, nested by each subject. For testing different hypotheses on the group effect, we followed the same procedure described above for mixed effects models.

## Supporting Information

S1 Dataset The individual data points of participants. As explained in the journal instruction, this file provides the individual data points of participants for circadian rhythms, sleep and actigraphy.
(XLSX)
